# DUXAP10 inhibition attenuates the proliferation and metastasis of hepatocellular carcinoma cells by regulation of the Wnt/β-catenin and PI3K/Akt signaling pathways

**DOI:** 10.1042/BSR20181457

**Published:** 2019-05-31

**Authors:** Kun Han, Chunqi Li, Xin Zhang, Liang Shang

**Affiliations:** 1 Department of Gastroenterology, Xi’an Central Hospital, Xi’an 710003, China; 2 Department of Medicine, School Hospital of Xi’an International Studies University, Xi’an 710128, China; 3 Department of Geriatrics, Shaanxi Proxincial People’s Hospital, Xi’an 710068, China

**Keywords:** β-catenin, DUXAP10, EMT, Hepatocellular carcinoma, PI3K/Akt

## Abstract

The long non-coding RNA DUXAP10 has been involved in the development, progression, and metastasis in several human cancers, but its biological function and underlying mechanism in hepatocellular carcinoma (HCC) still undetermined. The present study was proposed to explore the effect of DUXAP10 on the growth and metastasis of HCC cells and the potential mechanisms involved. The results showed that DUXAP10 is dramatically elevated in HCC tumor tissues and cell lines. Knockdown of DUXAP10 by DUXAP10 si-RNA significantly inhibited the cell viability, proliferation and induce the apoptosis of HCC cell line. Meanwhile, inhibition of DUXAP10 attenuates the cell migration, invasion, and epithelial–mesenchymal transition (EMT) process. No significant change of JNK MAPK pathway was detected in DUXAP10 siRNA transfected HCC cell lines. The β-catenin and pAkt levels were decreased in the Hep G2+DUXAP10 siRNA and SMMC7721+DUXAP10 siRNA groups, while the activation of Wnt/β-catenin or PI_3_K/Akt suppressed the inhibition of DUXAP10 siRNA on cell proliferation and migration. Collectively, DUXAP10 plays a critical role in regulating HCC development, potentially by regulating EMT and cell proliferation through the PI_3_K/Akt and Wnt/β-catenin signaling. Inhibition of DUXAP10 in HCC HepG2 cells could attenuate the EMT and cell proliferation and invasion. Therefore, DUXAP10 might be a promising therapy target to inhibit the growth of HCC.

## Introduction

Hepatocellular carcinoma (HCC) is one of the most aggressive cancers due to its high rate of recurrence [1,2]. It is diagnosed as an advanced stage, more importantly, with a poor median survival rate from 6 to 20 months [3]. It was well established that the primary cause responsible for the failure of traditional treatments is the unlimited proliferation, metastasis, and the abilities in the heterogeneous lineages of cancer cells. Thus, further investigation into the mechanism that regulates the proliferation is important for developing novel therapies to improve the survival rate in HCC patients.

In the past decade, the significance of long non-coding RNA (lncRNA) has been identified [5–8]. These tens of thousands of IncRNAs play a plethora of roles in diverse pathological processes, especially in carcinogenesis. For instance, the lncRNA DUXAP8 helps the gastric cancer cell proliferation and tumor genesis. The lncRNA RSU1p2 is significantly up-regulated in cervical cancer [7]. DUXAP10 was found to be overexpressed in bladder cancer, esophageal cancer, gastric cancer, liver HCC, and colorectal cancer [7]. Moreover, results from DUXAP10 loss-and-gain-of-function assays illustrated that DUXAP10 overexpression is in line with gastric cancer patients’ poor prognosis. Knockdown of DUXAP10 significantly decreased the proliferation, migration, and invasion of cells in gastric cancer. Recently, study indicated that the overexpression of lncRNA DUXAP10 is associated with the prognosis of HCC patients. Nevertheless, the biological functions and underline mechanisms of DUXAP10 in HCC have not been revealed. Thus, the present study is aimed to explore the role of DUXAP10 in HCC and the potential mechanisms involved.

## Materials and methods

### Tissue samples

We obtained tumor tissues and adjacent non-tumor tissues from 32 advanced HCC patients, from the Xi’an Central Hospital. The diagnosis standard for advanced HCC was from the current management of HCC: an eastern perspective, etc. [9,10]. The informed consent was obtained from all patients, and the study was approved by the research Ethics Committee of Xi’an Central Hospital. The research has been carried out in accordance with the World Medical Association Declaration of Helsinki and a stated ethics body.

### Cell culture

HCC cell lines, including Hep G2, SMMC7721, and normal HCC cell line LO2, were all purchased from Chinese Academy of Science (Cell Biology of Shanghai Institute, China). Cells were cultured in DMEM culture medium (Gibco, U.S.A.), 100 U/ml penicillin and streptomycin 10% fetal bovine serum (Gibco) were added [7,11]. All the cells were incubated in a humidified environment at 37°C with 5% CO_2_ supply.

### RNA extraction and quantitative real-time PCR

Trizol was used for the isolation of total RNA from the tumor tissues and adjacent non-tumor tissues. The quantitative real-time PCR was performed using a PrimeScript™ RT-PCR Kit (Takara, Japan) on a Roche LightCycler480-II real-time thermal cycler (Roche). In brief, 45 μl reaction solutions containing 50 ng cDNA, 25 μl of FastStart Universal SYBR Green Master, 0.5 μl of forward primer and reverse primer, and 19 μl PCR-grade water were subjected to 95°C for 10 min and then 40 cycles of 95°C for 15 s, 56°C for 60 s, and 68°C for 45 s. The primers used were as follows: DUXAP10 Forward primer 5′-GGTTCAACAGTATGGCTCCAAAG-3′ Reverse primer 5′-GACTGCCCATCCACAGATGAAG-3′; GAPDH was used as the reference gene. RT-PCR was conducted at 95°C for 3 min, 95°C for 3 s, 60°C for 30 s, 40 cycles. 2^−ΔΔ*C*^_T_ method was used for calculation, and each sample was examined in triplicate.

### SiRNA interference

Hep G2, SMMC7721 cells were cultured 6 × 10^4^/well in a six-well plate, and transfected with DUXAP10-siRNA and negative control-RNA (GenePharma, China). The transfection was performed according to the manufacturer’s protocol of lipofectamine 2000 (Invitrogen, Carlsbad, CA, U.S.A.). Total 6 h after transfection, the lipofectamine 2000-coated siRNA were all washed out with culture medium. And 48 h after transfection, cells were used for other experiments [12].

### Cell viability test

The MTT assay was used for cell viability evaluation. Cells were seeded to 96-well plates with 2000 cells/well and incubated for 1, 2, 3, 4, and 5 d. In the MTT assay, in each group, 20 μl 5 mg/ml, MTT (Sigma–Aldrich, U.S.A.) solution was added to each well and then the cells were continue to incubate at 37°C for 4 h. After that, the MTT solution was removed, and 200 µl of DMSO was added for each well. After 15 min incubating in 37°C, the plate was measured by a microplate reader at 490 nm (BioTek Instruments, U.S.A.).

### Cell proliferation test

For BrdU binding, remove the existing culture medium from the cells and replace with 10 µM BrdU labeling solution (Abcam, U.S.A.). Incubate the cells in the BrdU labeling solution at 37°C for 12 h and then immunofluorescence staining was used. Fix cells using 4% paraformaldehyde in 0.M PBS for 15 min at room temperature. For sample permeabilization, using 0.3% Triton X-100 for 15 min. For DNA hydrolysis, incubate cells in 1 M HCL for 10 min at 37°C. Then incubate cells with 3% BSA for 20 min to block the possible unspecific binding of the antibodies. Incubate cells with diluted BrdU-antibody (Abcam, U.S.A.) in 1% BSA overnight at 4°C. Then incubate cells with secondary antibody Cy3-goat anti-mouse (Abcam, U.S.A. and mounting the slides with Mountain medium with DAPI (Blue). Images were collected from at least three independent cultures per group were used for statistical analysis [11]. For CCK-8 assay, cells were seeded to 96-well plates with 2000 cells/well and incubated for 1, 2, 3, 4, and 5 d. Total 10 µl of CCK-8 solution was added to each well of the 96-well plate. Then incubate the plate for 2 h in the incubator. Measure the O.D. at 450 nm using a microplate reader.

### Migration and invasion experiment

Transwell membranes (8 μm pore, 6.5 mm diameter; Corning, NY, U.S.A.) were used to detect cell migration and invasion. Before the experiment, SMMC7721 and Hep G2 were suspended separately and cultured in the serum-free DMEM culture medium for 24 h. After that, they were plated (1 × 10^5^ cells/well) in the top chamber of the inserts cultured in the serum-free DMEM. Then, in the lower chamber of the well, 500 μl of 10% serum-containing DMEM were added. The cells were allowed to migrate at 37°C for 24 h and then removed from the upper chamber using cotton swabs. For invasion experiment, cells were seeded on Matrigel-coated (1:5 Dilute, 50 μl/well, BD Biosciences) membrane inserts (1 × 10^4^/well). Cells that successfully migrated to the underside of the membrane were stained with 0.1% crystal violet and counted in four randomly selected fields by using a light microscope.

### Cell apoptosis analysis

The Annexin V and FITC-propidium iodide (PI) double staining assay (BD Biosciences, U.S.A.) were used to estimate the apoptotic cells. Briefly, 48 h of post-DUXAP10 siRNA transfection cells were harvested and stained with 5 µl of FITC-Annexin V and 5 µg/ml of PI. After incubation for 15 min in the dark, the cells were detected by flow cytometry. The Annexin V positive and PI negative cells are in the apoptotic stage; the double negative cells are considered alive; the double positive cells are dead cells.

### Western blotting analysis

Total protein was extracted using RIPA (Beyotime, China), and the concentrations were determined by BCA kit (Beyotime, China). Total 10 μg of samples were loaded and separated by 10–12% SDS-PAGE gels. Proteins on the gel were transferred to PVDF. After blocking membrane in 10% skim milk, the membrane was incubated in primary antibody overnight at 4°C. Following by incubating with the HRP-conjugated goat anti-rabbit (Santa Cruz Biotechnology, U.S.A.) secondary antibodies for 2 h at room temperature. The membranes were visualized with ECL systems (Amersham Biosciences, Chalfont St. Giles, U.K.). GAPDH was used to normalize the data. The primary antibodies used: E-cadherin (Abcam, U.S.A.), N-cadeherin (Abcam, U.S.A.), Vimentin (Abcam, U.S.A.), pAkt (CST, U.S.A.), Akt (CST, U.S.A.), pJNK (CST, U.S.A.), JNK (CST, U.S.A.), GAPDH (Proteintech, U.S.A.), and β-catenin (Santa Cruz Biotechnology, U.S.A.) [13–15].

### Statistical analysis

Data were processed using SPSS13.0 software and expressed as means ± S.D. Differences amongst groups were assessed by one-way ANOVA; differences between groups were analyzed by *t* test. A *P*<0.05 was considered as statistically significant.

## Results

### DUXAP10 is up-regulated in advanced HCC

**Figure 1:**
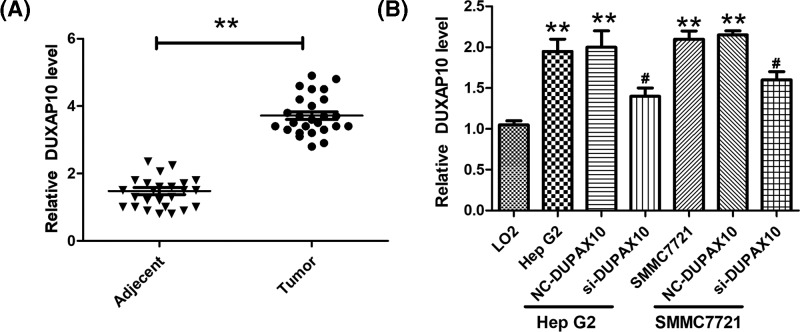
DUXAP10 is up-regulated in advanced HCC and HCC cell lines (**A**) The RNA level of DUXAP10 was examined by RT-PCR in tumor tissues of HCC patients (*n*=26) samples and paired adjacent non-tumor tissues (adjacent tissue, *n*=25) (**P<0.01 vs adjacent tissue). (**B**) The DUXAP10 RNA expression level in human HCC cell lines (Hep G2 and SMMC7721 cell lines) and normal human hepatic cell line (LO2) (**P<0.01 vs LO2 cell line; #*P*<0.05 vs Hep G2 and SMMC7721 cell lines).

The expression level of DUXAP10 in advanced HCC, 32 HCC tissues and normal adjacent tissues were dissected from HCC patients, and the mRNA expression of DUXAP10 was detected using RT-PCR analysis. Amongst these tissues, there are 25 normal adjacent tissues that express DUXAP10, and 26 tumor tissues that express DUXAP10. Our result showed that DUXAP10 expression level was significantly higher in HCC than in the adjacent tissues (Figure 1A). In order to study the mechanisms of the regulation of DUXAP10 in HCC development, Hep G2 and SMMC7721 cell lines were selected to reveal the mechanism of DUXAP10 in HCC. In addition, LO2, a normal human hepatic cell line was used as control. The RT-PCR result illustrated that, the DUXAP10 expression level in Hep G2 and SMMC7721 is significantly higher than LO2 cells (Figure 1B). By using DUXAP10 siRNA, the DUXAP10 RNA level was decreased significantly (Figure 1B).

### DUXAP10 si-RNA could inhibit the viability, proliferation, and induce apoptosis of HCC cells

**Figure 2:**
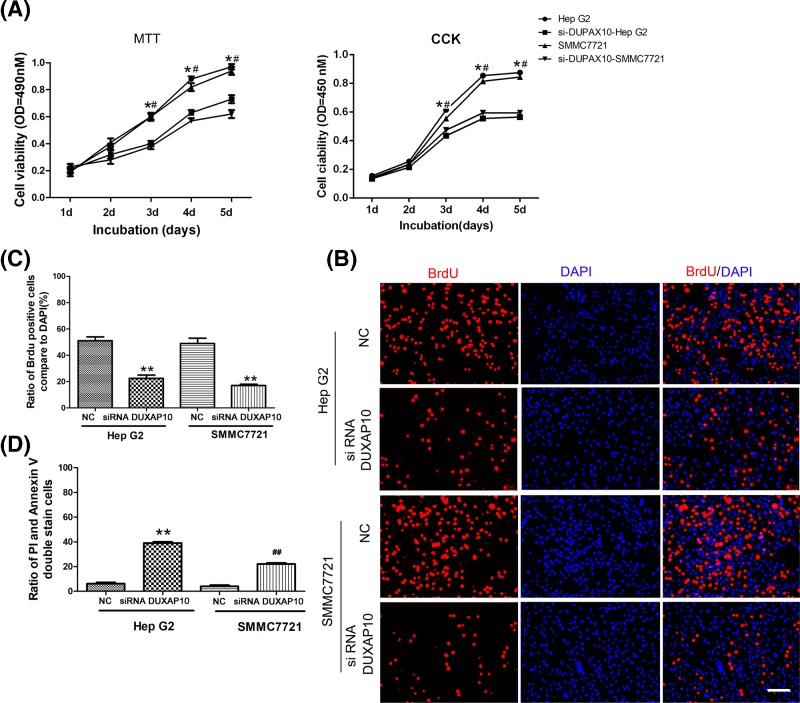
DUXAP10 si-RNA could inhibit the viability, proliferation and induce the cell apoptosis of HCC cell line (**A**) Cell was incubated for 1–5 days and then the cell viability was examined by MTT and CCK essay (*P<0.05 Hep G2 vs si-DUXAP10-Hep G2; #*P*<0.05 SMMC7721 vs si-DUXAP10-SMMC7721). (**B**) Cell proliferation was examined by BrdU assay; cells were incubated with BrdU (Red) for 12 h, and DAPI (Blue) was used to mark the nucleus, scale bar = 50 μm. (**C**) Cell proliferation rate was calculated by dividing the number of BrdU positive cells to the total cell number (DAPI positive cell numbers). (**D**) The Annexin V and FITC-PI double staining assay were used to estimate the apoptotic cells. Total 48 h post-DUXAP10 siRNA transfection cells were harvested and stained with FITC-Annexin V and PI and detected by flow cytometry. The Annexin V positive and PI negative cells are in the apoptotic stage; the double negative cells are considered alive (**P<0.01 Hep G2 vs si-DUXAP10-Hep G2; *##P*<0.01 SMMC7721 vs si-DUXAP10-SMMC7721).

MTT and CCK assay were used for the cell viability study. The results showed that the HCC cell line viability is higher than the normal hepatic cell LO2. However, by suppressing the DUXAP10, the cell viability was decreased significantly compare with the control group (Figure 2A). Besides, the cell proliferation levels increased dramatically in the Hep G2 and SMMC7721 cells and decreased by inhibiting the DUXAP10 (Figure 2B,C). Furthermore, the FCM results illustrated that the inhibition of DUXAP10 could induce Hep G2 and SMMC7721 cell apoptosis (Figure 2D).

### DUXAP10 si-RNA inhibited HCC metastasis and epithelial–mesenchymal transition

**Figure 3:**
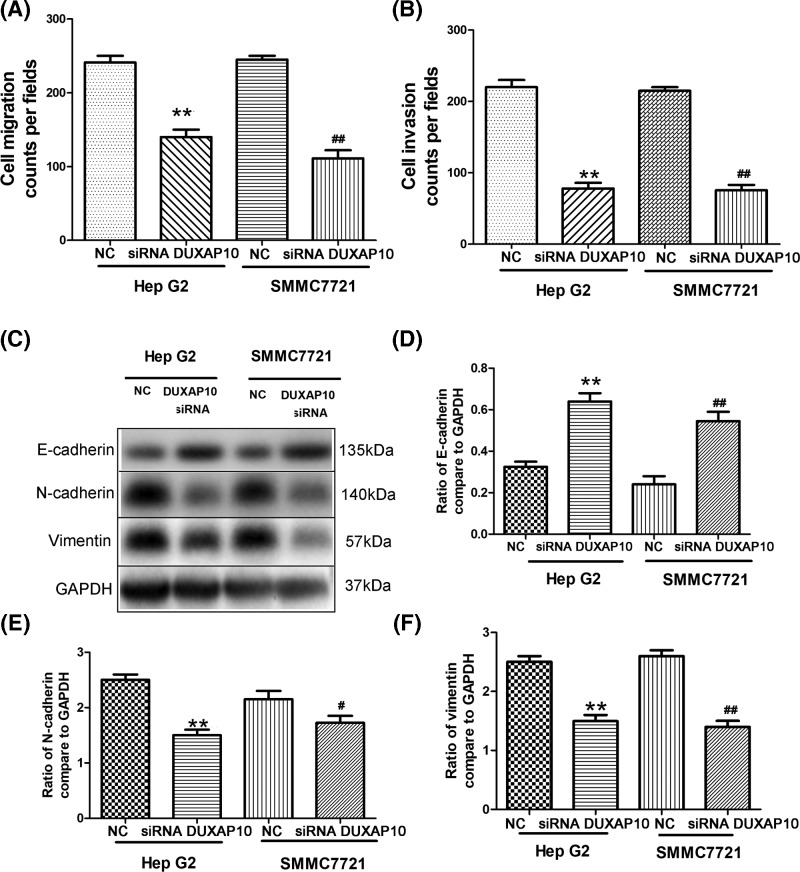
DUXAP10 si-RNA inhibited HCC metastasis and epithelial–mesenchymal transition Inhibition of DUXAP10 attenuates the cell migration, invasion, and EMT process (**A,B**). Transwell assays were used to determine cell migration and invasion status of HCC cells. Cells were plated in the top chamber of the inserts and cultured in the serum-free DMEM. Total 500 μl of 10% serum-containing DMEM were added in the lower chamber of the well. For migration test, the cells were allowed to migrate for 24 h and then removed from the upper chamber. Cells were seeded on Matrigel-coated membrane inserts for invasion experiment. Cells appeared in the underside of the membrane were stained with crystal violet and counted (**P<0.01 Hep G2 vs si-DUXAP10-Hep G2; *##P*<0.01 SMMC7721 vs si-DUXAP10-SMMC7721). (**C**) EMT-related factors, the E-cadherin, N-cadherin, and vimentin, were determined by western blotting. (**D**) Ratio of E-cadherin to GAPDH has been calculated (**P<0.01 Hep G2 vs si-DUXAP10-Hep G2; *##P*<0.01 SMMC7721 vs si-DUXAP10-SMMC7721). (**E**) Ratio of N-cadherin to GAPDH has been calculated (**P<0.01 Hep G2 vs si-DUXAP10-Hep G2; *#P*<0.05 SMMC7721 vs si-DUXAP10-SMMC7721). (**F**) Ratio of N-cadherin to GAPDH has been calculated (**P<0.01 Hep G2 vs si-DUXAP10-Hep G2; *##P*<0.01 SMMC7721 vs si-DUXAP10-SMMC7721).

Next, the cell migration and invasion status of HCC cells were evaluated. HepG2 and SMMC7721 cells have higher migration and invasion ability compare with DUXAP10 siRNA treated group (Figure 3A,B). Next, the levels of epithelial–mesenchymal transition (EMT)-related factors, for instance, the E-cadherin, N-cadherin, and vimentin were analyzed. Our result illustrates that the inhibition of DUXAP10 increased the levels of E-cadherin (Figure 3C,D). The DUXAP10-siRNA decreased the protein expression level of N-cadherin and vimentin (Figure 3C,E,F).

### β-catenin and PI_3_K/Akt signaling pathway were involved in the regulation of DUXAP10 on HCC cell proliferation and migration

**Figure 4:**
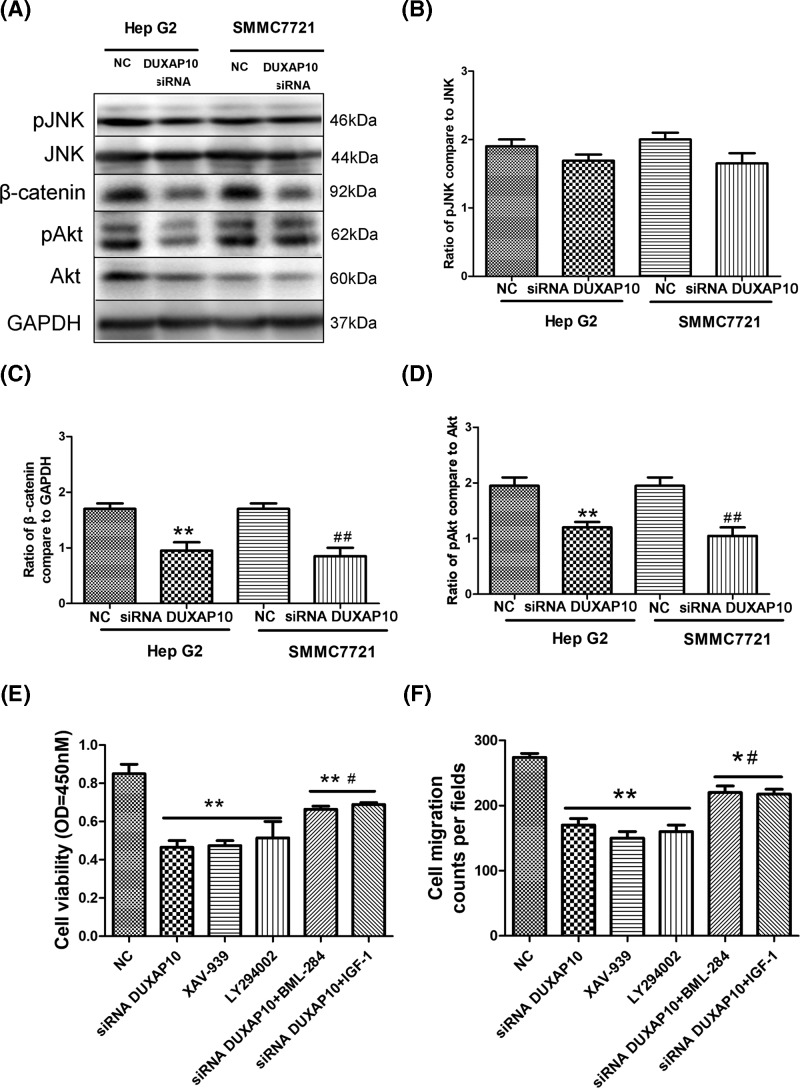
β-catenin and PI_3_K/Akt signaling pathway were involved in the regulation of DUXAP10 on HCC cell proliferation and migration (**A**) The GAPDH, pJNK/JNK, β-catenin, and pAkt/Akt level were determined using western blotting. (**B**) Ratio of pJNK/JNK; (**C**) Ratio of β-catenin to GAPDH (**P<0.01 Hep G2 vs si-DUXAP10-Hep G2; *##P*<0.01 SMMC7721 vs si-DUXAP10-SMMC7721). (**D**) Ratio of pAkt/Akt (**P<0.01 Hep G2 vs si-DUXAP10-Hep G2; *##P*<0.01 SMMC7721 vs si-DUXAP10-SMMC7721). (**E**) Cell proliferation was examined using CCK-8 assay in Hep G2 cells (**P<0.01 vs NC; *#P*<0.05 vs si-DUXAP10, XAV-939, LY294002). (**F**) Cell migration was determined using Transwell assay (**P<0.01 vs NC; *P<0.05 vs NC; *#P*<0.05 vs si-DUXAP10, XAV-939, LY294002).

It was suggested that the JNK MAPK, Wnt/β-catenin, and PI_3_K/Akt signaling pathway were involved in the HCC cell migration and invasion. In the present study, we evaluated the phosphorylate-JNK (pJNK)/JNK, and Wnt/β-catenin, and phosphorylate-Akt/Akt level in the Hep G2, Hep G2+DUXAP10 siRNA, SMMC7721, SMMC7721+DUXAP10 siRNA groups. Western blotting data illustrate that there is no significant change of pJNK/JNK in Hep G2+DUXAP10 siRNA and SMMC7721+DUXAP10 siRNA groups. Indicating the DUXAP10 regulates the HCC cell migration and invasion through other pathways (Figure 4A,B). The β-catenin and pAkt levels were decreased in the Hep G2+DUXAP10 siRNA and SMMC7721+DUXAP10 siRNA groups (Figure 4A,C,D). We further used XAV-939, the inhibitor of Wnt/β-catenin, LY294002 the inhibitor of PI_3_K/Akt to determine whether Wnt/β-catenin and PI_3_K/Akt regulates the DUXAP10 induced cell migration and invasion. Our result illustrates that, by using the Wnt/β-catenin or PI_3_K/Akt inhibitor the cell proliferation and migration were decreased (Figure 4E,F). In addition, the activation of Wnt/β-catenin (activator: BML-284) or PI_3_K/Akt (activator: IGF-1), combined with DUXAP10 siRNA could suppress the inhibition of DUXAP10 siRNA on cell proliferation and migration (Figure 4E,F).

## Discussion

LncRNAs have diverse cellular functions such as cell proliferation and differentiation, and it has demonstrated that they are crucial regulators for a variety of tumor growth [11,15,16]. It was reported recently that 71.4% patients expressed higher DUXAP10 level than in non-tumor tissues in HCC tissues [5]. DUXAP10 was up-regulated in several HCC cancer cell lines, including SMMC-7721, MHCC-97H, Hep G2, Hep3B, and SK-Hep-1 cells [3,4,11]. Furthermore, DUXAP10 level in HCC tissues is significantly higher than the adjacent nontumor tissues. These results indicated that DUXAP10 is a risk factor for HCC. In addition, our results illustrate that the DUXAP10 level is more correlated with the severity of the HCC, especially in advanced HCC patients, they have a higher ratio of overexpressed DUXAP10. DUXAP10 is a risk factor for prognosis for HCC patients [4,5,12]. However, the mechanisms of DUXAP10 on the tumor growth in HCC patients remain unclear. The present study indicated that cell viability and proliferation were all significantly decreased by inhibiting the DUXAP10 in SMMC-7721 and in Hep G2 cell. Furthermore, the DUXAP10-siRNA could also induce SMMC-7721 and in Hep G2 cell apoptosis. This result agrees with the study in ovarian cancer in DUXAP10 [17]. Collectively, DUXAP10 is highly involved in the tumor cell growth of HCC. In addition, in advanced HCC patients, the ratio of HCC positive patient is higher than in the HCC patients (including the early stage and the advanced stages). This evidence indicated that DUXAP10 might be involved in the HCC cell migration and invasion.

It has been reported that DUXAP10 is significantly increased in non-small cell lung cancer tissues and knockdown of DUXAP10 could inhibit the inhibited non-small cell lung cancer cell migration and invasion. Moreover, the cell proliferation, migration, and invasion in non-small cell lung cancer cells are significantly up-regulated in the DUXAP10 overexpression cell model. The *in vivo* study showed that knockdown DUXAP10 could impair non-small cell lung cancer growth [12]. We illustrated that in HCC cells, the inhibition of DUXAP10 could attenuate the cell migration and invasion. Therefore, it can be proposed that the DUXAP10 regulates cell migration and invasion in the broader manner; not in a cancer type specific manner. Therefore, the EMT factors may also affect by the DUXAP10. In the present study, DUXAP10 siRNA could significantly increase the E-cadherin expression, while reducing the levels of Vimentin, indicating that EMT was inhibited. These findings were consistent with the migration and invasion results.

The JNK signal pathway plays an important role in the advance of HCC [18,19]. It was demonstrated that compound deficiency of JNK1/2 in hepatocytes do not prevent HCC development [18–20], which indeed suggests that JNK deficiency in hepatocytes increased the tumor. Our results suggested that after knockdown of DUXAP10, cell proliferation was inhibited and apoptosis was increased. Furthermore, the knockdown of DUXAP10 also inhibited PI_3_K/Akt signaling pathway [3,9,13,21]. Combining with these results, we found that by inhibiting DUXAP10 expression, the PI_3_K/Akt signaling has also attenuated. This suggested that PI_3_K/Akt is involved in the DUXAP10 related HCC cell proliferation and invasion. Moreover, Wnt/β-catenin is activated in the tissue of HCC patients, which indicates that Wnt/β-catenin is involved in HCC tumor growth. This is in parallel with our result. More importantly, we have proved that Wnt/β-catenin is taken part in the DUXAP10 related HCC cell proliferation [22].

In summary, our present study showed that DUXAP10 plays a critical role in regulating HCC development, potentially by regulating EMT and cell proliferation through the PI_3_K/Akt and Wnt/β-catenin signaling. Inhibition of DUXAP10 in HCC SMMC-7721 and Hep G2 cells could attenuate the EMT and cell proliferation and invasion. Therefore, DUXAP10 might be a potential target for overcoming therapy for HCC in the future.

## Abbreviations

CRC: colorectal cancer
EMT: epithelial–mesenchymal transition
HCC: hepatocellular carcinoma
lncRNA: long non-coding RNA
PI: propidium iodide
